# Bilateral Multiple Thoracic Disc Herniation Decompressed Through a Posterior Transpedicle Approach: A Case Report

**DOI:** 10.7759/cureus.100050

**Published:** 2025-12-25

**Authors:** Mohammed Awad Mohammed, Ahmed M. Sonbol, Farid Kassab, Meshal Altowairqi, Hassan Sirajaldeen Alhassan Ali, Mohammed M. Elgack, Saeed Alshwekany

**Affiliations:** 1 Orthopedic Surgery, Andalusia Group for Medical Services, Jeddah, SAU; 2 Orthopedic Surgery, Dr. Suliman Fakeeh Hospital, Jeddah, SAU; 3 Orthopedics, King Abdulaziz Specialist Hospital, Taif, SAU; 4 Orthopedic Surgery, King's College London Hospital, Jeddah, SAU

**Keywords:** 4-level thoracic decompression, diskectomy, dorsal spine, intervertebral disc displacement, laminectomy, multilevel thoracic decompression, multiple thoracic disc herniation, posterior transpedicular

## Abstract

A four-level thoracic disc prolapse is extremely rare, especially when surgical intervention is required. The level of the disc and the herniation's direction determine the surgical approach of selection. A 46-year-old male presented with a two-week history of severe, sudden back pain and weakness in his lower extremities. Clinical presentation was suggestive of thoracic radiculopathy and myelopathy. Magnetic resonance imaging confirmed four-level disc prolapses at the levels of T9-T10, T10-T11, and T11-T12, which were central and in both directions. Decompression and fixation were done through the posterior transpedicular approach. The surgical approaches for decompression of multiple thoracic disc herniations are not commonly discussed in the literature due to their rarity. Generally, the anterior trans-thoracic approach is preferred over the posterior approach for one or two-level herniation. The posterior transpedicular approach was chosen in this case because it is less invasive and avoids combined approaches. Also, the herniated discs were in four consecutive levels, and the herniations were in different directions. In cases of multiple thoracic disc herniations requiring surgery, the posterior transpedicular approach allows access to the discs from both the right and left sides, enabling safe and complete decompression while avoiding more invasive or combined approaches.

## Introduction

Disc herniation occurs in approximately 40-50 per 100,000 individuals, making it a relatively common condition. Thoracic disc herniation, by contrast, is extremely rare, with an estimated prevalence of 1 per 1,000,000 individuals [1]. A herniated or bulged disc may compress the neural elements [2]. The presentation and the diagnosis of thoracic disc herniation can be challenging because it can mimic other pathologies such as muscular chest wall pain, herpes zoster, gallstones, cholecystitis, and peptic ulcer [3]. Magnetic resonance imaging (MRI) is the preferred imaging modality for providing detailed assessment and accurate diagnosis of spinal pathology [4]. Management ranges from conservative measures to invasive surgical intervention, with the choice of surgical approach depending on the level of the disc and the direction of herniation [5-7]. Generally, the anterior trans-thoracic approach is preferred over the posterior approach for one or two-level herniation [5,8,9]. In this case, the presentation was deceptive, and the herniated discs were bilateral and at four levels. The surgical approach chosen was less invasive, and all the pathologies were approachable. This case report is noteworthy due to the limited literature discussing surgical approaches for multiple thoracic disc herniations requiring decompression.

## Case presentation

A 46-year-old male presented with a two-week history of severe, sudden back pain and weakness in his lower extremities, inability to walk, and numbness involving his legs. The neurological examination revealed an inability to overcome gravity in the testable myotomes of the levels of L2, L3, L4, L5, and S1 of the bilateral lower extremities. Sensory testing for crude and fine touch, pain, and temperature was unremarkable bilaterally. Reflex examination showed hyperreflexia at the patellar tendon with the presence of clonus.

**Figure 1:**
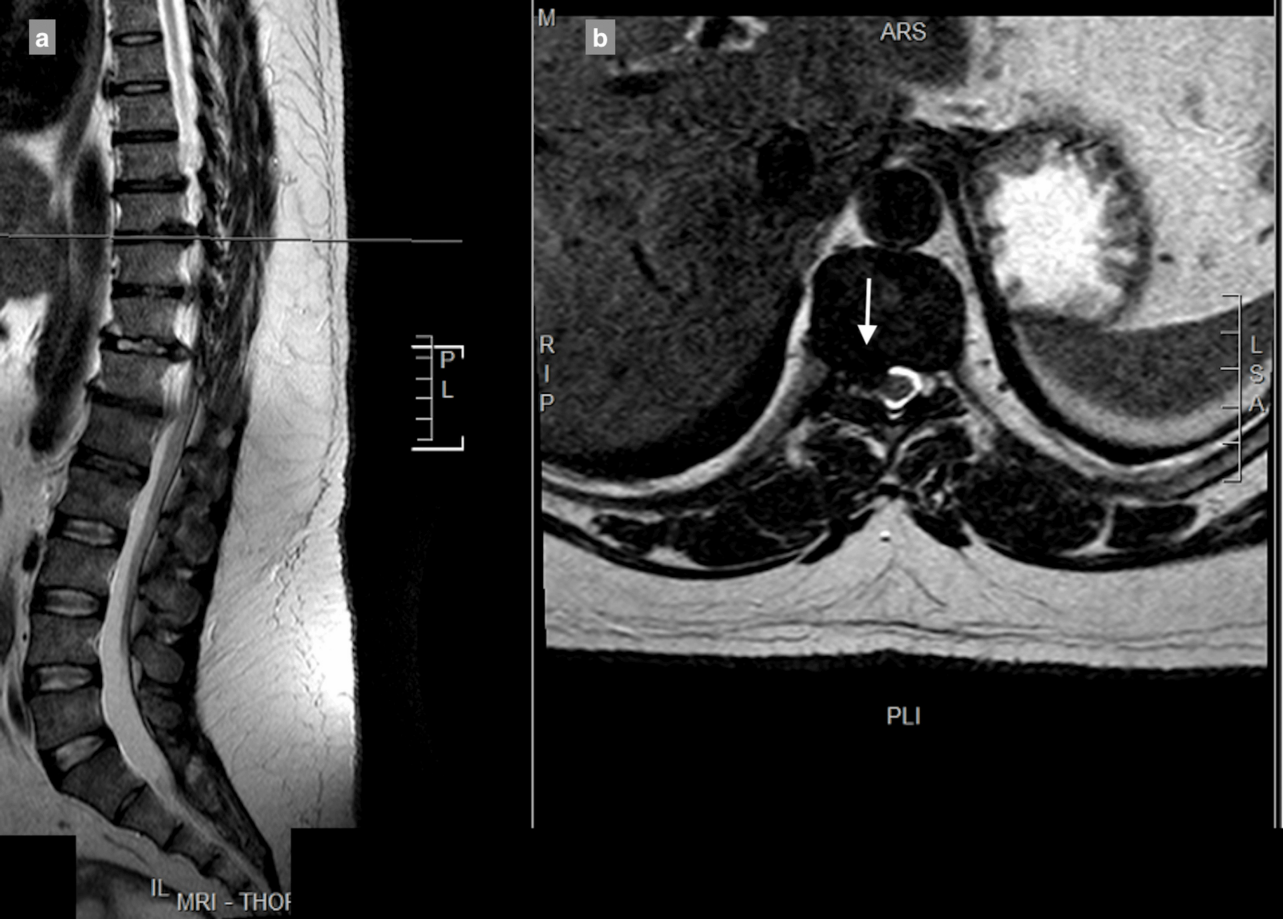
9–T10 central and right-sided disc extrusion causing spinal canal stenosis with compression of the spinal cord: (a) T2-weighted sagittal view; (b) T2-weighted axial view.

**Figure 2:**
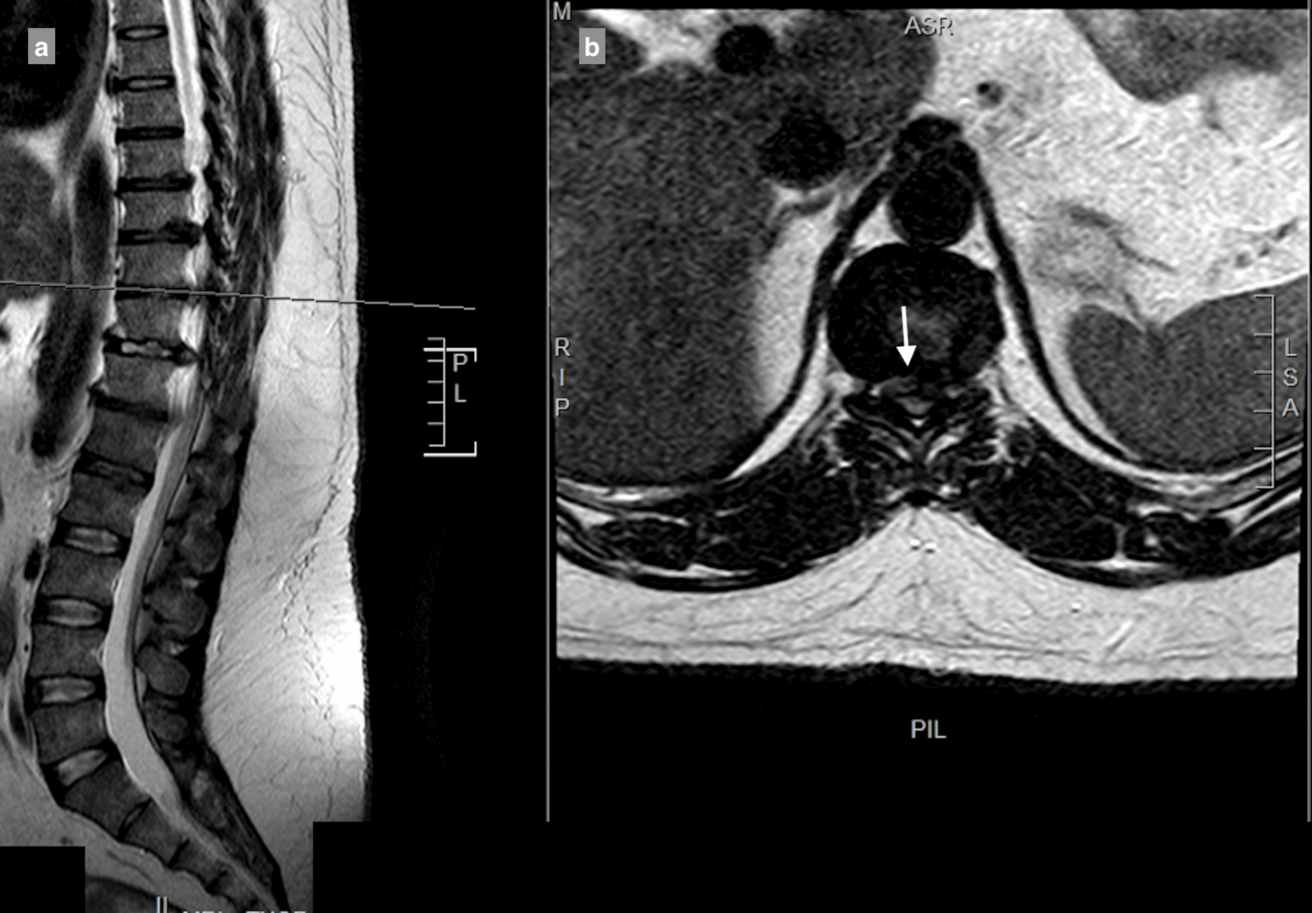
T10-T11 central disc prolapse causing spinal canal stenosis, with compression of the spinal cord: (a) T2 sagittal view, (b) T2 axial view.

**Figure 3:**
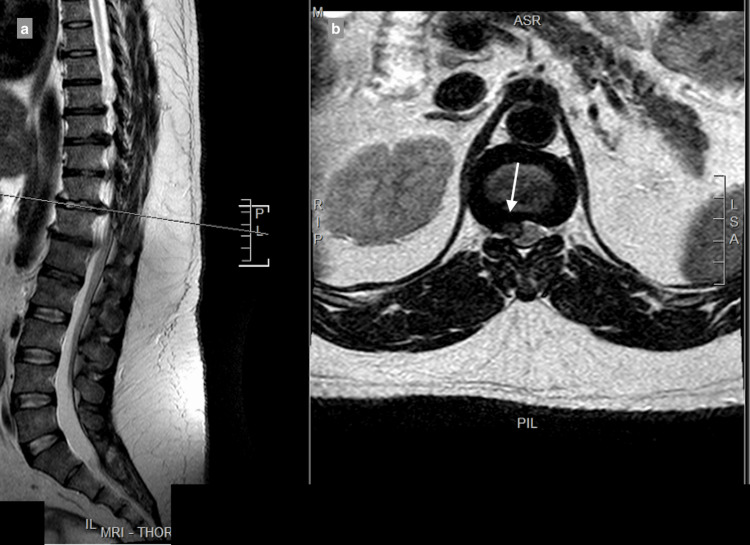
T11-T12 central disc and right-sided disc extrusion causes spinal canal stenosis, with compression of the spinal cord: (a) T2 sagittal view, (b) T2 axial view.

**Figure 4:**
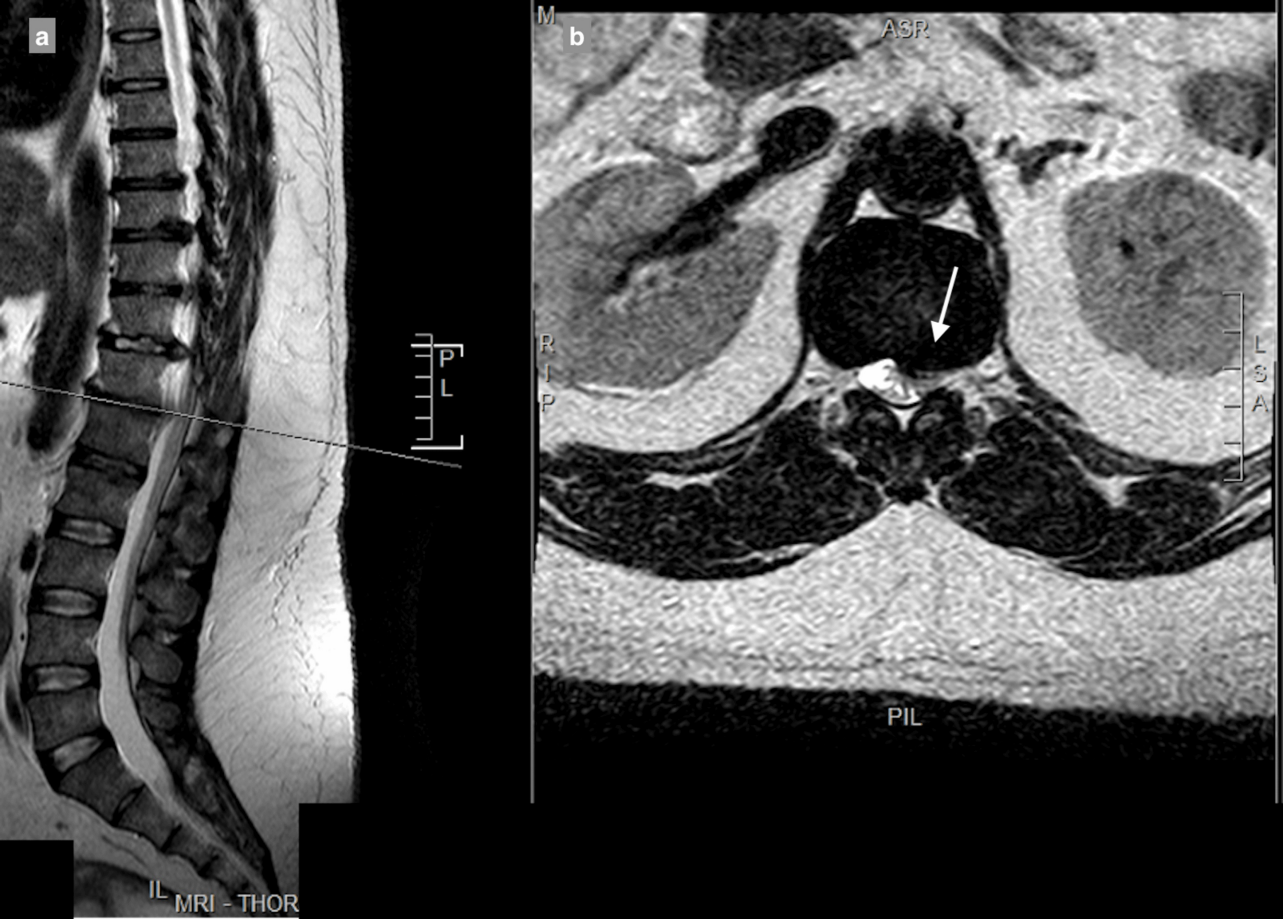
Central and left-sided T12–L1 disc prolapse causing spinal canal stenosis, with compression and a fanning appearance of the spinal cord: (a) T2 sagittal view, (b) T2 axial view.

MRI showed a central and right-sided disc prolapse at the T9-T10 level, a central disc prolapse at the T10-T11 level, a central and right-sided disc prolapse at the T11-T12 level, and a central and left-sided disc prolapse at the T12-L1 level, all causing cord compression at multiple levels (Figure 1-4).

Preoperative planning

A thoracotomy was considered for disc removal because the T10-T11 herniation was central and difficult to access via a posterior approach, whereas the remaining levels were amenable to posterior decompression. However, performing a thoracotomy would have required a thoracolumbar frenotomy to access T12-L1, which would not have facilitated decompression of the upper levels. Additionally, the disc herniations were located on both the right and left sides, potentially necessitating bilateral access, further limiting the suitability of a thoracotomy. Based on these considerations, a posterior transpedicular approach was selected.

Surgical technique and preparation

The patient was placed in a prone position on the Wilson frame. Motor evoked potentials were continuously monitored intraoperatively. A midline posterior approach was employed, with exposure of the bilateral laminae and facets from T9 to L2. Instrumentation was placed as follows: bilateral pedicle screws at T9, a unilateral left pedicle screw at T10, T11 was skipped, a unilateral left pedicle screw at T12, a unilateral right pedicle screw at L1, and bilateral pedicle screws at L2.

**Figure 5:**
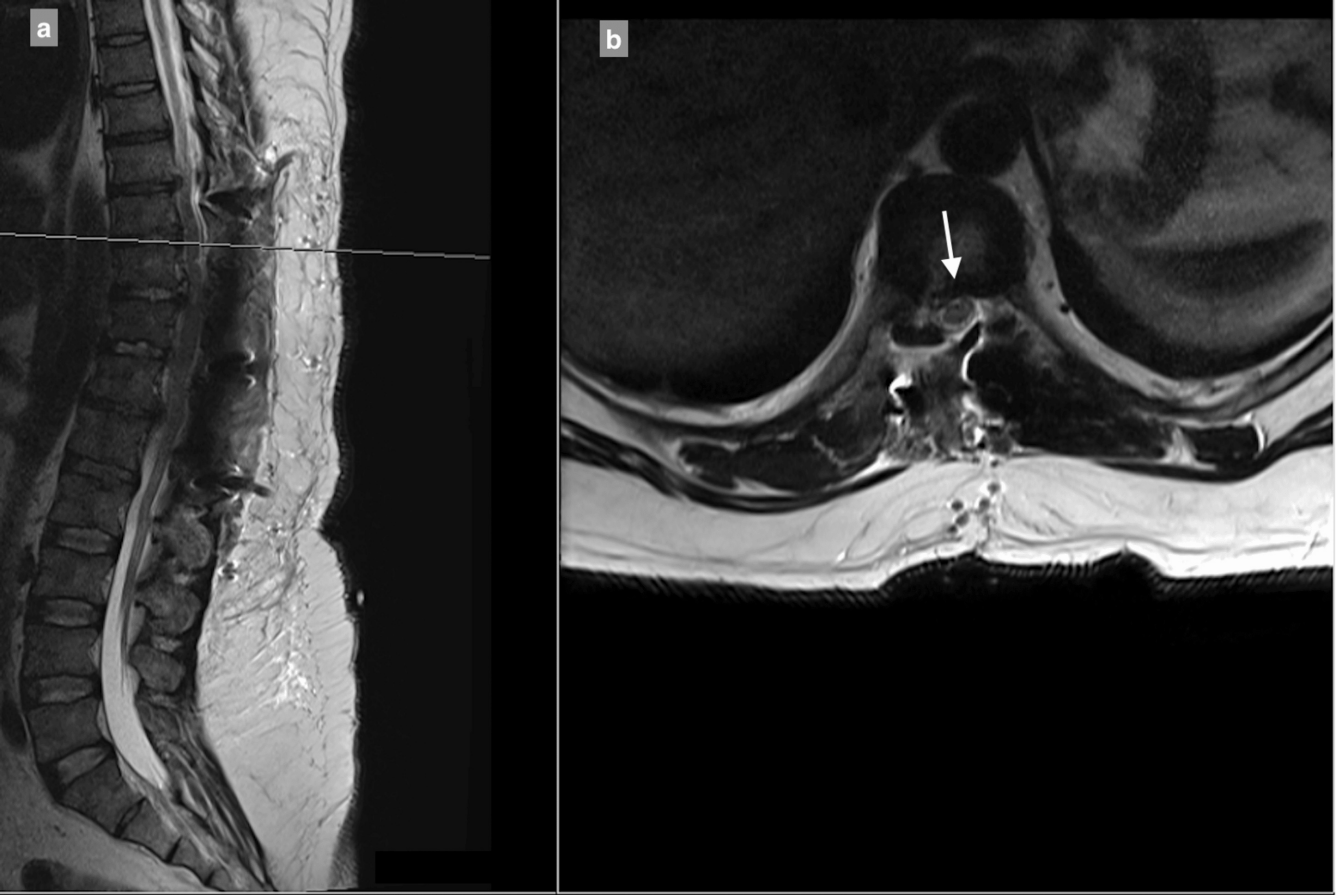
Postoperative MRI of T9-T10 showing the decompressed spinal cord with no apparent stenosis or collection: (a) T2 sagittal view, (b) T2 axial view.

**Figure 6:**
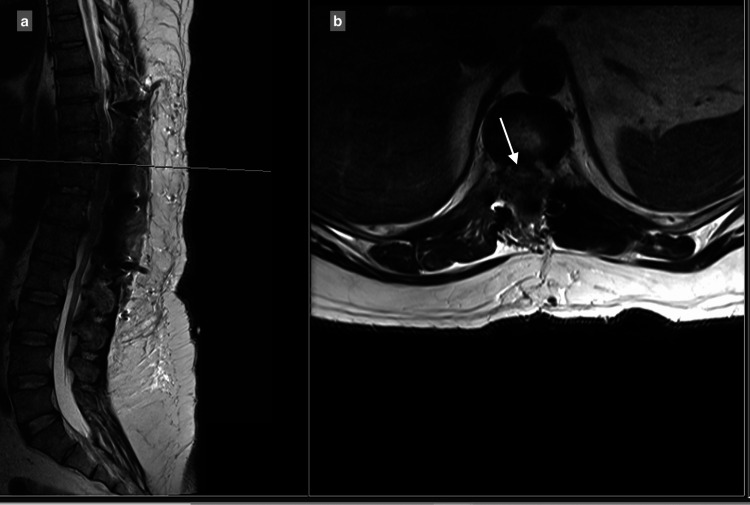
Postoperative MRI of T10-T11 showing the decompressed spinal cord with no apparent stenosis or collection: (a) T2 sagittal view, (b) T2 axial view.

**Figure 7:**
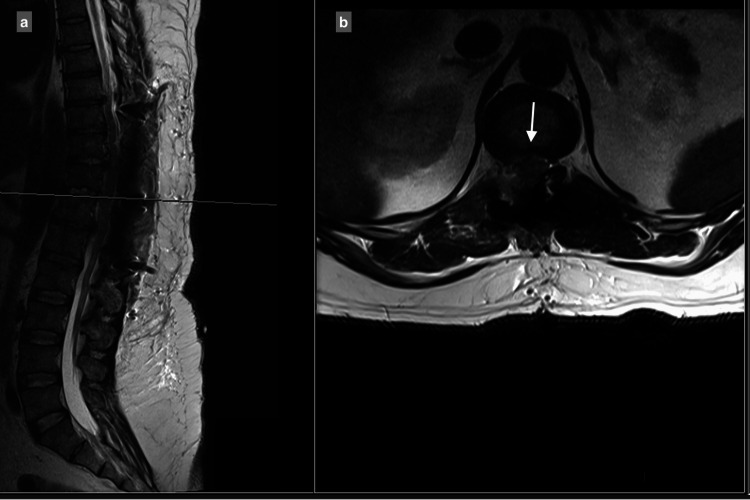
Postoperative MRI of T11-T12 showing the decompressed spinal cord with no apparent stenosis or collection: (a) T2 sagittal view, (b) T2 axial view.

**Figure 8:**
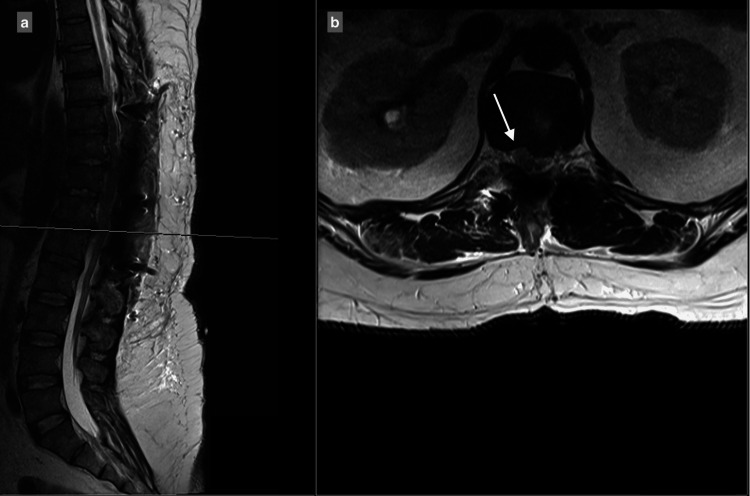
Postoperative MRI of T12-L1 showing the decompressed spinal cord with no apparent stenosis or collection: (a) T2 sagittal view, (b) T2 axial view.

**Figure 9:**
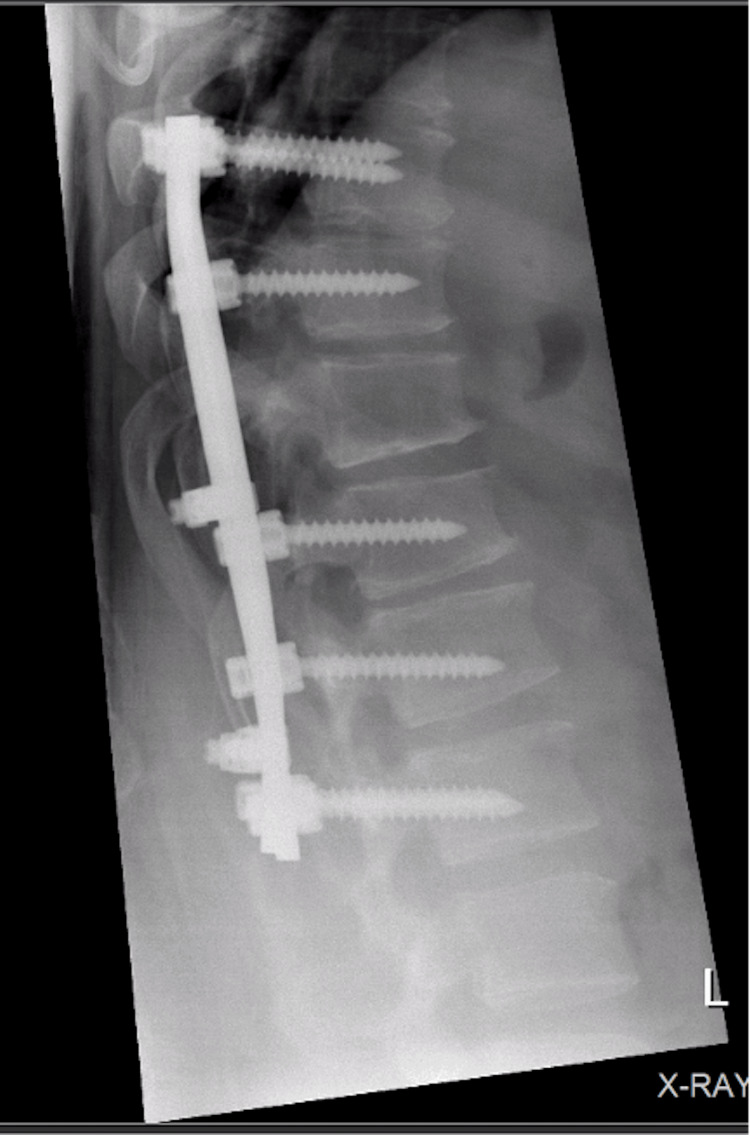
Postoperative X-ray lateral view showing good alignment with no loosening of the fused levels.

**Figure 10:**
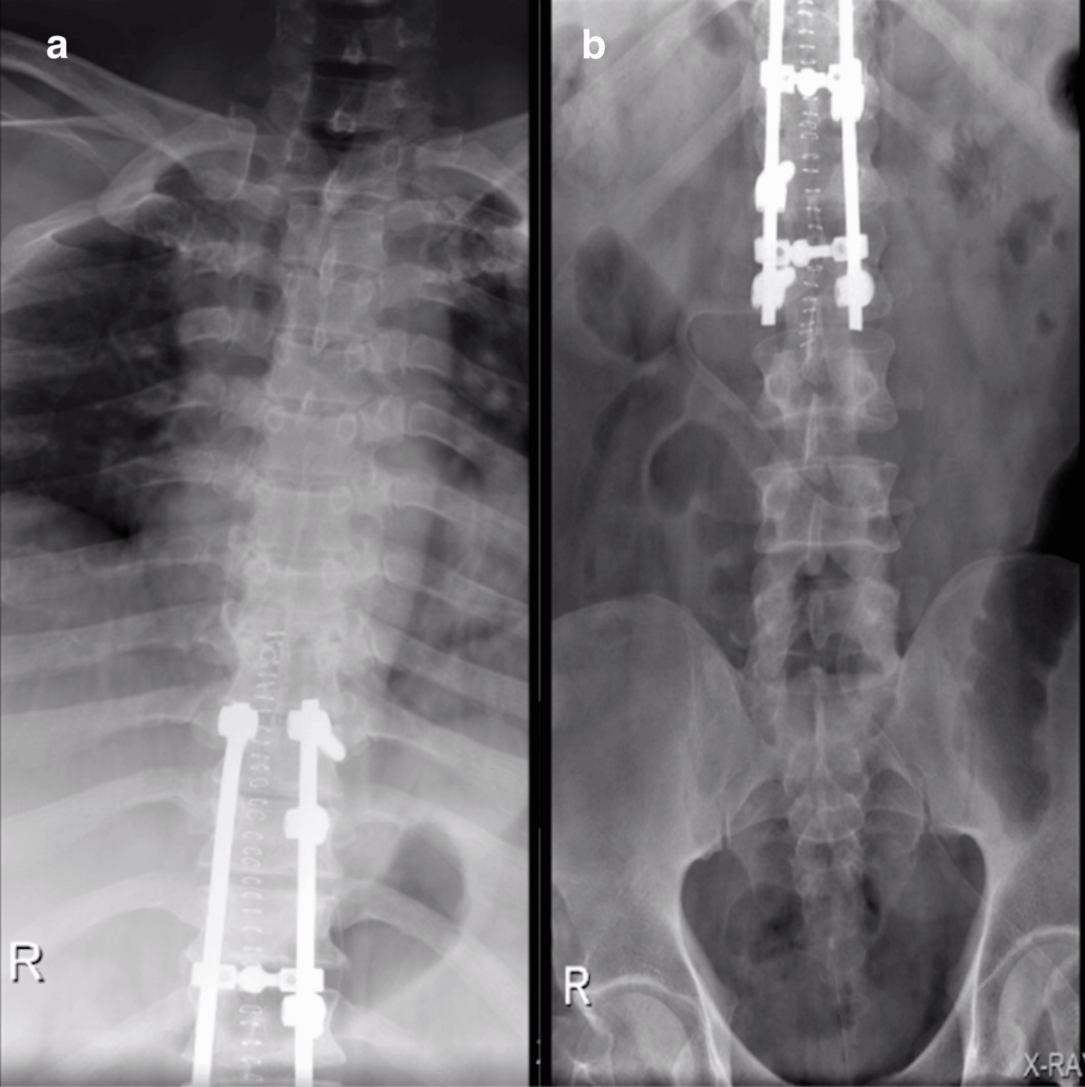
Postoperative X-ray A/P view showing good alignment with no loosening of the fused levels: (a) Thoracic spine (b) Thoraco-lumbar junction and lumbar spine.

The decompression process commenced with specific actions at varying vertebral levels. At the T12-L1 level, a hemilaminectomy was conducted on the left to perform a foraminectomy. By moving laterally to the cord, the bulging disc was identified, excised, and completely removed. Moving on to the T11-T12 level, a hemilaminectomy was carried out on the right side, and the bulging disc was removed through a laminectomy procedure without affecting the cord. Progressing to the T10-T11 level, a complete laminectomy was undertaken, and bilateral laminectomies were performed to remove the disc from both sides. A hooked instrument was utilized to reach the center and excise the bulging disc entirely. Subsequently, at the T9-T10 level, a hemilaminectomy was performed on the right side, alongside the foraminectomy, to remove the bulging disc without impacting the cord. Connecting rods were then introduced between T9 and L2 and secured using transverse rod connectors. Decortication was performed with autograft material to facilitate fusion. During the procedure, all discs were noted to be soft, and no calcification was encountered. Following the surgery, post-operative X-ray and MRI scans revealed satisfactory decompression and fixation, as depicted in Figures 5-10.

The postoperative course was uneventful, with no change in neurological status during the three-day hospital stay. At the 2-month follow-up, following a structured physical therapy regimen, the patient exhibited a 30% increase in quadriceps muscle strength and a marked improvement in gait stability and balance during weight-bearing activities. He was walking normally with some deficit in light touch and pain sensations in the feet. At the 8-month follow-up, the patient has regained his baseline lower extremity strength and reports experiencing only mild back discomfort.

## Discussion

Multilevel and bilateral thoracic disc herniations are rare, with an estimated annual incidence of approximately 1 per million individuals, and account for a small proportion of all disc herniations. Surgical intervention involving multiple levels is uncommon and remains inadequately addressed in the literature, which is largely limited to case reports and small clinical studies focusing on diagnostic considerations, surgical approaches, and patient outcomes [4,10].

Stillerman et al. followed 82 patients with thoracic disc herniation who underwent surgical decompression [11]. The choice of surgical approach for each patient depends on the position of the herniated disc, with the transthoracic approach being the most commonly used. Other surgical approaches include the transfacet pedicle-sparing, lateral extracavitary, and transpedicular techniques. Bohlman et al followed 19 patients with thoracic disc herniation who needed surgery [6]. Decompression was performed via an anterior transthoracic approach, which was found to carry a risk of spinal cord injury. Dickman et al. concluded that the anterior transthoracic approach is preferred for large central herniations, calcified discs, or transdural disc herniations, as posterior approaches in these settings carry a high risk of neurological injury [7]. Currier et al. performed 19 transthoracic discectomies and fusions, and they found that the procedure was safe. Conversely, laminectomies were commonly associated with neurologic deterioration [8]. While the transthoracic approach provides superior exposure for lower thoracic disc lesions, it was linked to higher morbidity and complication rates. In contrast, the transpedicular posterior approach affords ample exposure coupled with decreased surgical duration and blood loss, facilitating prompt mobilization and discharge [12]. The long-term outcomes of the posterior approach were comparable to those of the transthoracic approach [13].

In this case, the decision to utilize a posterior transpedicular approach was based on its minimally invasive nature. Moreover, given the presence of herniated discs across four contiguous levels with bilateral involvement, a combined approach would have required a more extensive surgical procedure. The herniated discs had no evidence of calcification, adhesions, or transdural penetration. Adequate access was achieved to decompress right-central, central, and left-central herniated discs. Obstacles, like disc adhesions, calcifications, and intradural penetration, may affect the decompression through the posterior approach and could lead to poor outcomes.

## Conclusions

For multiple thoracic disc herniations requiring surgery, the posterior transpedicular approach provides bilateral access to the discs, allowing safe and adequate decompression while avoiding more invasive or combined approaches. It is important to note that factors such as disc adhesions, calcifications, and intradural penetration may limit decompression and contribute to suboptimal outcomes with this approach.
